# Aquatic ecosystem responds differently to press and pulse nutrient disturbances as revealed by a microcosm experiment

**DOI:** 10.1002/ece3.9438

**Published:** 2022-10-22

**Authors:** Yu Zhao, Rong Wang, Enlou Zhang, Baohua Guan, Min Xu

**Affiliations:** ^1^ State Key Laboratory of Lake Science and Environment Nanjing Institute of Geography and Limnology, Chinese Academy of Sciences Nanjing China; ^2^ University of Chinese Academy of Sciences Beijing China

**Keywords:** climate change, press disturbance, pulse disturbance, resistance, submerged macrophyte

## Abstract

Due to climate change and increasing anthropogenic activities, lakes are disturbed frequently, usually by press (e.g., diffused pollution, rising temperatures) or pulse (e.g., storms, rainfall, pollution events) disturbances. Both press and pulse disturbances can affect abiotic and biotic environments, changing the structure of ecosystems and affecting ecosystem services. To confront with the effects of climate change and increasing anthropogenic activities, understanding the different effects of press and pulse disturbances on lake ecosystems is essential. This study assessed the effect of press and pulse disturbances of phosphorus on a microcosmic aquatic ecosystem by measuring the total phosphorus (TP), algae density, and physiological indicators of submerged macrophytes. We found that the microcosmic aquatic ecosystem responded differently to press and pulse disturbances. Our results suggested that it had a lower resistance to pulse phosphorus disturbances than to press phosphorus disturbances. There were significantly higher nutrient concentrations and algal densities in the pulse treatment than in the press treatment. Positive feedback was found between the biomass of submerged macrophytes and the water quality. There was a higher submerged macrophytes biomass at low TP concentration and algal density. In the context of climate change, press and pulse disturbances could have severe impacts on lake ecosystems. Our findings will provide some insight for further research and lake management.

## Graphical abstract

The differences in aquatic ecosystem responses to press and pulse disturbances were experimentally investigated. Pulse disturbances were more stressful than press disturbances to submerged macrophytes.

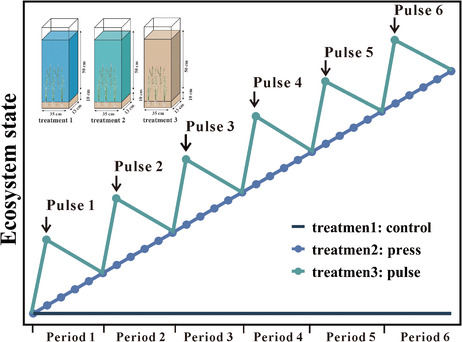

## 1 INTRODUCTION

In recent decades, human activities and global changes have triggered changes in ecosystems, leading to problems such as climate warming, water pollution, and biodiversity loss, both regionally and globally (Greaver et al., 2016; Halpern et al., 2019; Zhang et al., 2020). According to the Intergovernmental Panel on Climate Change (IPCC) 2014 report, extreme events will increase in the 21st century (Field et al., 2014) and ecosystems are more likely to be exposed to different disturbances (Papalexiou & Montanari, 2019; Wentz et al., 2007). For example, the increasing water temperatures caused by climate change may lead to the release of phosphorus from sediments, thereby increasing algal biomass (Kosten et al., 2011). Extreme events such as storms can increase the frequency of short‐term nutrient pulses in lakes, thus altering the nutrient balance and disrupting the ecosystem structure of the lakes (Kasprzak et al., 2017; Leigh et al., 2015).

Disturbances can be classified as press or pulse disturbances (Glasby & Underwood, 1996; Jentsch & White, 2019; Yang et al., 2008). Press disturbances are lasting or continuous disturbances that are usually a result of anthropogenic activities (Glasby & Underwood, 1996). Press disturbances such as nutrient input from agricultural runoff can threaten drinking water and endanger human health through pathogen transfer (Zhang et al., 2021). Pulse disturbances are short‐term, relatively discrete events that are usually the result of physical forces (storms or floods), chemical inputs (nutrients, antibiotics), or bioturbation (invasion of exotic species) (Brasell et al., 2021). Pulse disturbances in the form of resources can lead to dramatic changes in ecosystems by facilitating large increases in the numbers of primary producers and altering food web structures and ecosystem stability (Carpenter et al., 1999; Holt, 2008; Scheffer et al., 2001). Press and pulse disturbances can occur simultaneously in the context of climate change, and the superimposed effects of different disturbances could have catastrophic consequences for ecosystems (Beniston et al., 2007; Easterling et al., 2000).

Lake ecosystems, which can serve as sensitive indicators of environmental changes, provide critical ecosystem services and functional values such as biodiversity conservation, habitat formation, and nutrient transformation (Heino et al., 2009; O'Beirne et al., 2017; Tolonen et al., 2014). Due to global warming, an increase in extreme events, and cultural eutrophication, it is essential to understand the impact of press disturbances (e.g., surface source pollution, rising temperatures) and pulse disturbances (e.g., floods, storms, pollution events) on lake ecosystems. Press disturbances from herbicide use have been shown to promote increases in lake turbidity and phytoplankton biomass, leading to alterations in top‐down control processes by diminishing zooplankton populations (Rumschlag et al., 2020). Pulse disturbances such as nutrients input and heatwaves in lakes could exacerbate the incidence of harmful algal blooms and change the distribution patterns of plants (Deegan et al., 2012; Higashino & Stefan, 2014; Jeppesen et al., 2009; Johnk et al., 2008).

Submerged macrophytes and algae play significant roles in the functioning of shallow lakes, and their interaction affects the function and stability of lake ecosystems (Sayer et al., 2010). The survival of submerged macrophytes can be threatened by algae through shading and algal toxins (Jia et al., 2016). Eutrophication caused by nutrient input has altered the relationship between submerged macrophytes and algae in shallow freshwater lakes, resulting in the shift from a macrophyte‐dominated clear‐water state to a phytoplankton‐dominated turbid‐water state (Hargeby et al., 2007; Scheffer et al., 1993, 2001). The nutrient inputs, which are likely to become more frequent due to climate change and increased human activities, can allow algal blooms and the consequent loss of submerged macrophytes in many shallow lakes (Deegan et al., 2012; Johnk et al., 2008). It is interesting to know whether such aquatic ecosystems composed of submerged macrophytes and algae respond differently to press/pulse disturbances of nutrients. To answer this question, aquatic ecosystems consisting of submerged macrophytes and algae were constructed through a microcosm experiment and were exposed to different forms of nutrient disturbances. Three treatments were set up: (1) no nutrient addition (control treatment), (2) a constant input of nutrients (press treatment), and (3) the same amount of nutrients as (2) but as pulse additions (pulse treatment). Our object is to identify the differences in the response of aquatic systems to press and pulse nutrient disturbances and the mechanisms responsible for the differences. The study will provide some insight for further research and lake management.

## 2 MATERIALS AND METHODS

### 2.1 Experimental design

We used nine experimental aquariums (35 cm length, 13 cm width, 65 cm height, and 29.575 L). The water level was maintained at 50 cm (22.75 L), and evaporation losses were compensated once a week. Three treatments were set up: (1) no nutrient addition (control treatment), (2) a constant input of nutrients (press treatment), and (3) the same amount of nutrients as (2) but as pulse additions every 5 days (pulse treatment). Each treatment had three replicates. The water used for the experiments was distilled water. The sediment was 10 cm thick, taken from Lake Taihu, and filtered through a 1 mm aperture sieve.

**FIGURE 1:**
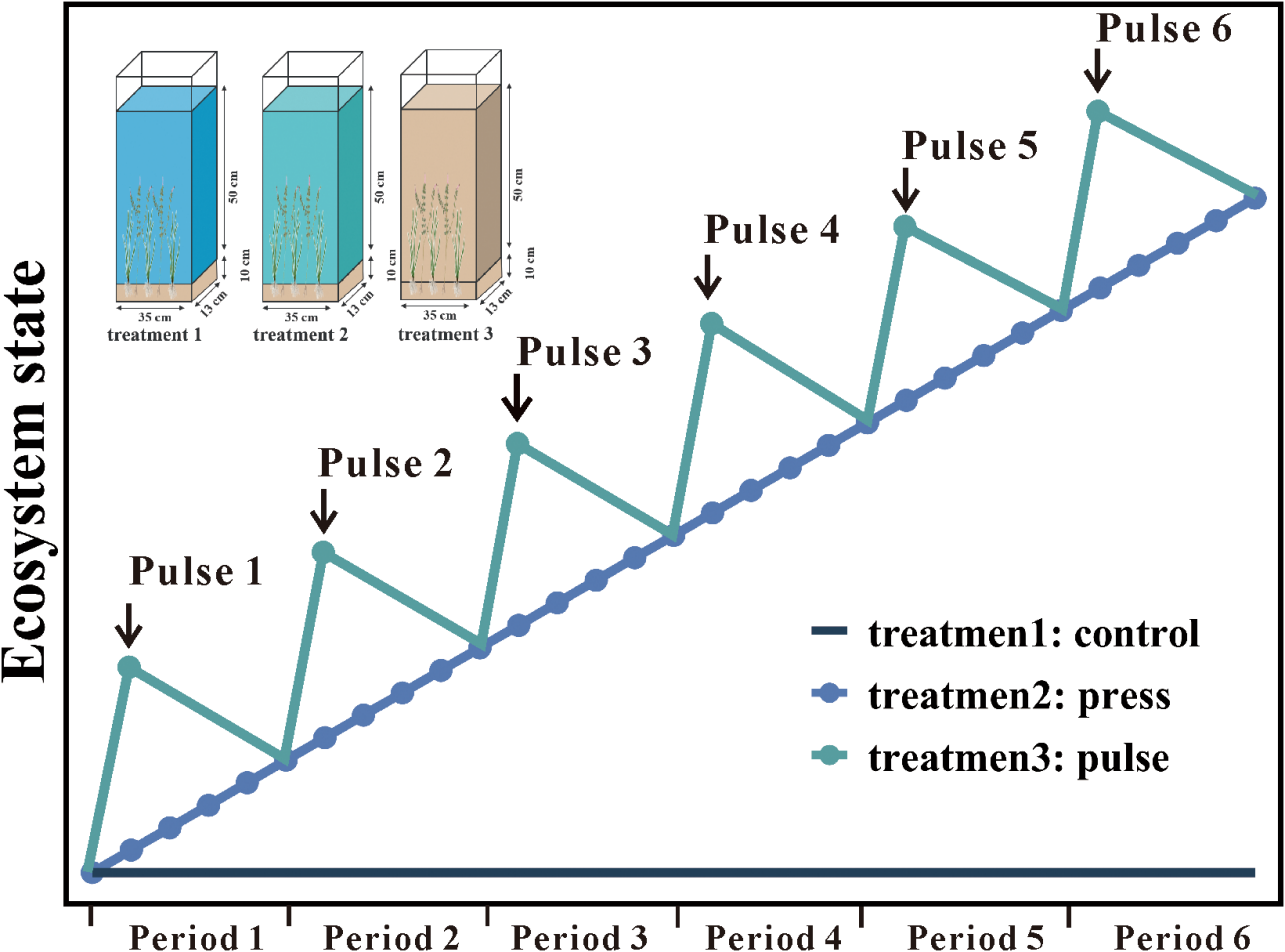
Schematic diagram of the experimental setup. The experiments involved control (treatment 1), press (treatment 2), and pulse treatments (treatment 3). The press treatment consisted of a constant addition of BG11 every day, and the pulse treatment consisted of a pulse addition of BG11 every 5 days. The ecosystem state on the vertical axis can be represented by the concentration of nutrients, the population of organisms, and other indicators.

The nutrient variable observed in the water column was total phosphorus (TP), added in the form of a BG11 culture medium (Rippka et al., 1979). The initial TP concentration of all the treatments in the water column was 0.014 ± 0.002 mg L^−1^. We set the TP concentration in the water column at the end of the experiment to be the same for the press and pulse treatments, both at 0.3 mg L^−1^. The difference between the two treatments was the method of nutrient addition, with the constant addition of BG11 every day for the press treatment and a pulse addition of BG11 every 5 days for the pulse treatment. The experiment was conducted over 30 days which contained six periods of 5 days each (Figure 1).

Two types of submerged macrophytes, typical and widely distributed in freshwater lakes of middle and lower reaches of the Yangtze River, were planted in all three treatments: the meadow‐forming submerged macrophyte *Vallisneria natans* and the canopy‐forming submerged macrophyte *Myriophyllum spicatum* (Zhong et al., 2013). The plants were sourced from Lake Taihu. Submerged macrophytes of uniform morphology were selected and gently washed for measurement and planting. The water quality was maintained in optimal clear states when the biomass proportions of *V. natans* were between 60% and 80% in the communities (Figure S1). Therefore, the biomass proportion of *V. natans* was set at 70%. The total fresh weight of the submerged macrophytes was 15 g, consisting of 10.5 g of *V. natans* and 4.5 g of *M. spicatum*. Then, the submerged macrophytes were transplanted into aquariums and acclimatized for 7 days. The initial TP concentration and OD680 were 0.014 ± 0.002 mg L^−1^ and 0.003 ± 0.001, respectively. The aquariums were placed in a light incubator with an illumination level of 12,000 lx for 16 h at 25°C during the day and 0 lx for 8 h at 22°C during the night (Szabo et al., 2022).

### 2.2 Sampling and processing

Water samples 20 cm below the water surface were collected once a day in all treatments. TP concentrations in the water column were measured using combined persulfate digestion (Ebina et al., 1983). The optical density 680 (OD680) value, which represents the algal density, was measured at a wavelength of 680 nm using an ultraviolet–visible spectrophotometer accurately and rapidly (Poulickova et al., 2008).

After 30 days, intact plants were collected and washed for laboratory determination of indicators. Each individual plant was carefully collected, and the epiphyton was separated from the macrophyte using a small brush in the laboratory. Filamentous algae were collected with a scraper from the walls of the aquariums and dried naturally on foil. Weight and root/shoot were expressed in fresh weight.

Peroxidase (POD), catalase (CAT), and superoxide dismutase (SOD) were extracted from fresh *V. natans* tissues. The enzyme content of *M. spicatum* was not analyzed, due to insufficient fresh weight for grinding. POD activity was determined by an increase in absorbance at a wavelength of 470 nm due to guaiacol oxidation (Civello et al., 1995). The unit of CAT activity was defined as the amount of enzyme that decomposed 1 μmol H_2_O_2_ per minute (Candan & Tarhan, 2003). SOD activity was measured using the method described by Ewing and Janero (1995).

We obtained 3 ml water samples (20 cm below the water surface) from the experimental aquariums each day. The samples were dark‐acclimated for 15 min. The minimum fluorescence (*F*_0_) value and the maximum fluorescence (*F*_m_) value were measured by a Phyto‐PAM (Walz). The difference between the *F*_m_ and *F*_0_ values is the variable fluorescence (*F*_v_). Then, the maximum photochemical efficiency of photosystem II (*F*_v_/*F*_m_) was measured. *F*_v_/*F*_m_ reflects the maximum photosynthetic capacity of a plant, and its value decreases when plants are light‐limited (Woitke et al., 2004).

### 2.3 Data analysis

The diffuse attenuation coefficient was expressed as light attenuation at the surface and 40 cm underwater. The photon flux densities at the surface and 40 cm underwater were measured by an Apogee MQ‐510 underwater PAR meter (Apogee Instruments, Inc.; Chen et al., 2016). The diffuse attenuation coefficient reflects the transparency of the water. The higher the value is, the more turbid the water. The value of *K*_d_ was calculated as follows:

(1) $$K_{d}=\frac{\ln I_{0}-\ln I_{40}}{Z}$$

where *K*_d_ (m^−1^) is the diffuse attenuation coefficient at 40 cm underwater; *I*_0_ (μmol m^2^ s) is the photon flux density at the surface; *I*_40_ (μmol m^2^ s) is the photon flux density at 40 cm underwater; and *Z* is the depth (m) (Liu et al., 2013).

Resistance is the ability to withstand disturbance (Folke et al., 2010; Nimmo et al., 2015). In our study, resistance was expressed as the response of the TP concentration in the water column to nutrient addition. The calculation of resistance was adapted from previous research (Jentsch & White, 2019; Yi & Jackson, 2021) and was defined as follows:

(2) $$\text{Resistance}=-\frac{{\mathrm{\Delta TP}}_{\text{measured}\;\left(\text{actual value}\right)}}{{\mathrm{\Delta TP}}_{\text{theoretical}}}$$

where ΔTP_measured (actual value)_ refers to the actual change in measured TP of the experimental system following the addition of BG11, and ΔTP_theoretical_ refers to the value of the expected change in TP. When ΔTP_measured (actual value)_ is <0, it indicates that adding BG11 did not increase the TP concentration and the system could return to the pre‐disturbance state. The opposite is true when ΔTP_measured (actual value)_ is >0. The higher the resistance, the more tolerant the system is to nutrient additions.

The total effect, which represents the magnitude of the disturbance effect, is defined as the area under the curve for TP concentration (Jentsch & White, 2019). We calculated the total press effect and the total pulse effect for each period. The initial pulse rate was defined as the change in TP concentration after pulse addition per unit time:

(3) $$\text{Initial pulse rate}=\frac{\mathrm{\Delta TP}}{\Delta t}$$

where ΔTP is equal to the TP concentration after the addition of BG11 minus the concentration before the addition. In this study, Δ*t* was 1. The smaller the initial pulse rate, the more resistant the system is to nutrient addition.

Locally weighted regression was used to smooth the temporal data. Locally weighted regression is a process of dividing a sample into small intervals, fitting a polynomial to the samples in the interval, and repeating the process to obtain a weighted regression curve for different intervals. Plots were constructed for R with a 95% confidence level for all analyses (Pacheco, 2011). Values are shown as mean ± standard error. The differences in the indicators between the treatments were evaluated by one‐way ANOVA at a significance level of 0.05. Before performing one‐way ANOVA, Shapiro–Wilk test was used to check normality, and Levene's test was used to check homoscedasticity. Non‐normal data were log‐transformed to obtain normality. Post hoc multiple comparisons of treatment means were performed by Tukey's least significant difference procedure at a significance level of 0.05. Pearson's correlations analysis was used to test for relationships between the biomass proportions of *V. natans* and *M. spicatum*. Normality was tested before the correlation analysis. The method used to normalize the data was the *z*‐score.

## 3 RESULTS

### 3.1 Water quality

**FIGURE 2:**
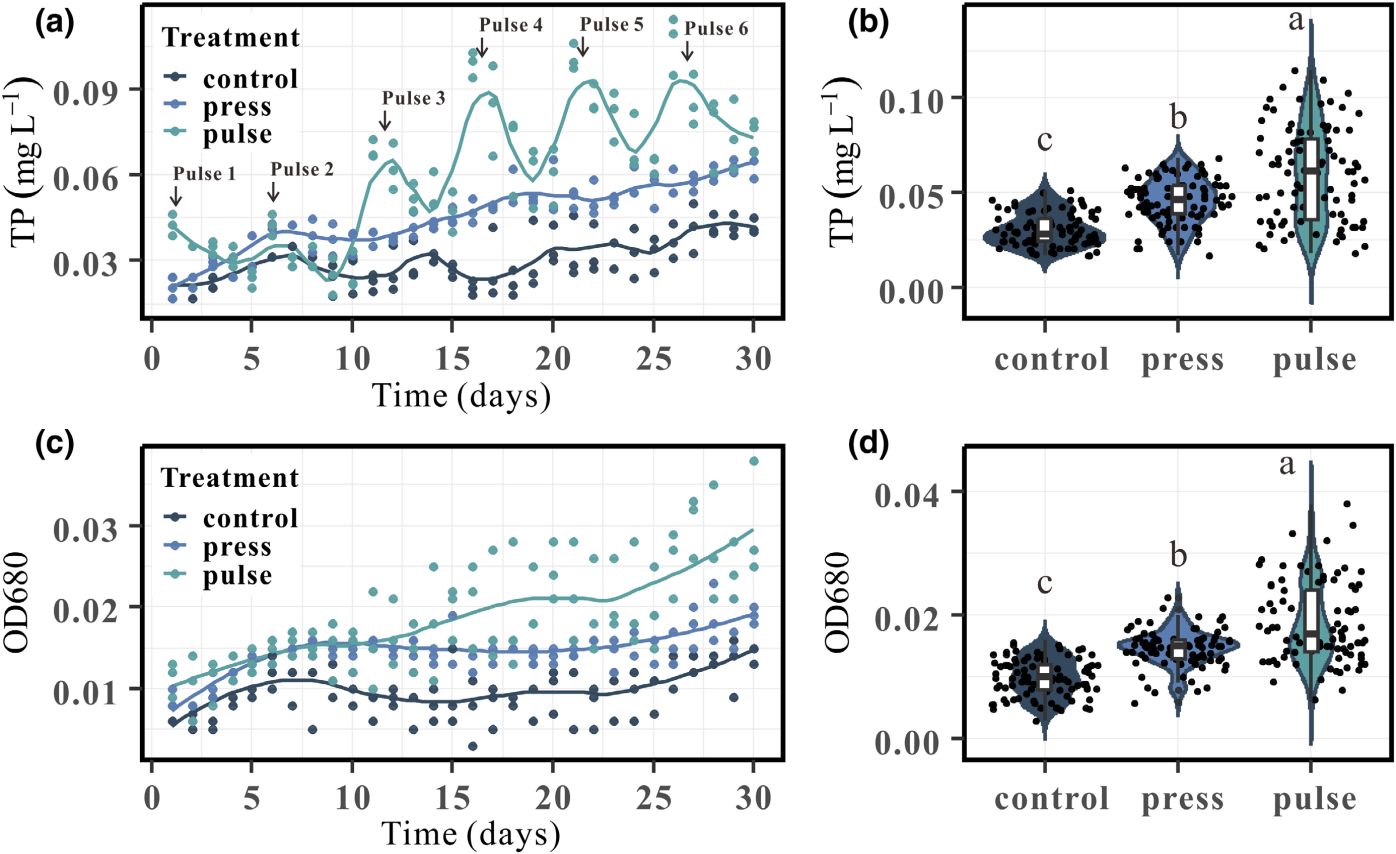
Variation in total phosphorus (TP) concentration (a) and OD680 (c) in the water column over time for different treatments. The curves are drawn by loess regression (locally weighted regression). Violin plots of TP (b) and OD680 (d) for the different treatments. The lowercase letters at the top of the violin plots indicate significant differences obtained by Tukey's least significant difference test at *p* < .05.

The TP and OD680 significantly differed between the treatments (*p* < .05; Table S1). Overall, the TP concentration was the greatest (0.06 ± 0.01 mg L^−1^) in the pulse treatment, at an intermediate level in the press treatment (0.05 ± 0.01 mg L^−1^), and the lowest in the control treatment (0.03 ± 0.02 mg L^−1^) (Figure 2b). The TP concentration in the water column of the pulse treatment initially increased and then declined during each period (Figure 2a). Overall, an increasing trend was found for the TP concentration in the press treatment. During the first period (day 1–5), the TP concentration in the pulse treatment decreased to a lower level than that in the press treatment. During the second period (day 6–10), the TP concentration in the press treatment decreased slightly but was still higher than that in the pulse treatment. Ten days later, the TP concentration in the pulse treatment was higher than that in the press treatment at all times, although the TP concentration in the pulse treatment gradually decreased after each disturbance. The OD680 value was the greatest (0.019 ± 0.006) in the pulse treatment, at an intermediate level in the press treatment (0.015 ± 0.003), and the lowest in the control treatment (0.100 ± 0.003) (Figure 2d). Throughout the experiment, the TP concentration and OD680 for the same treatment showed similar trends, indicating that the TP concentration may influence algal growth (Figure 2a,c). TP and OD680 were significantly lower in the press treatment than in the pulse treatment (*p* < .05; Table S1).

The turbidity of the water column varied with different treatments, with the clear state showing a smaller diffuse attenuation coefficient (*K*_d_). The mean value of *K*_d_ for the control treatment was 1.76 ± 0.05 m^−1^, which was smaller than the value of 2.22 ± 0.28 m^−1^ for the press treatment and the value of 2.52 ± 0.05 m^−1^ for the pulse treatment. The treatment with a high algal density had a high *K*_d_ value, indicating that algae can block light penetration and decrease transparency.

### 3.2 Changes in aquatic organisms

**FIGURE 3:**
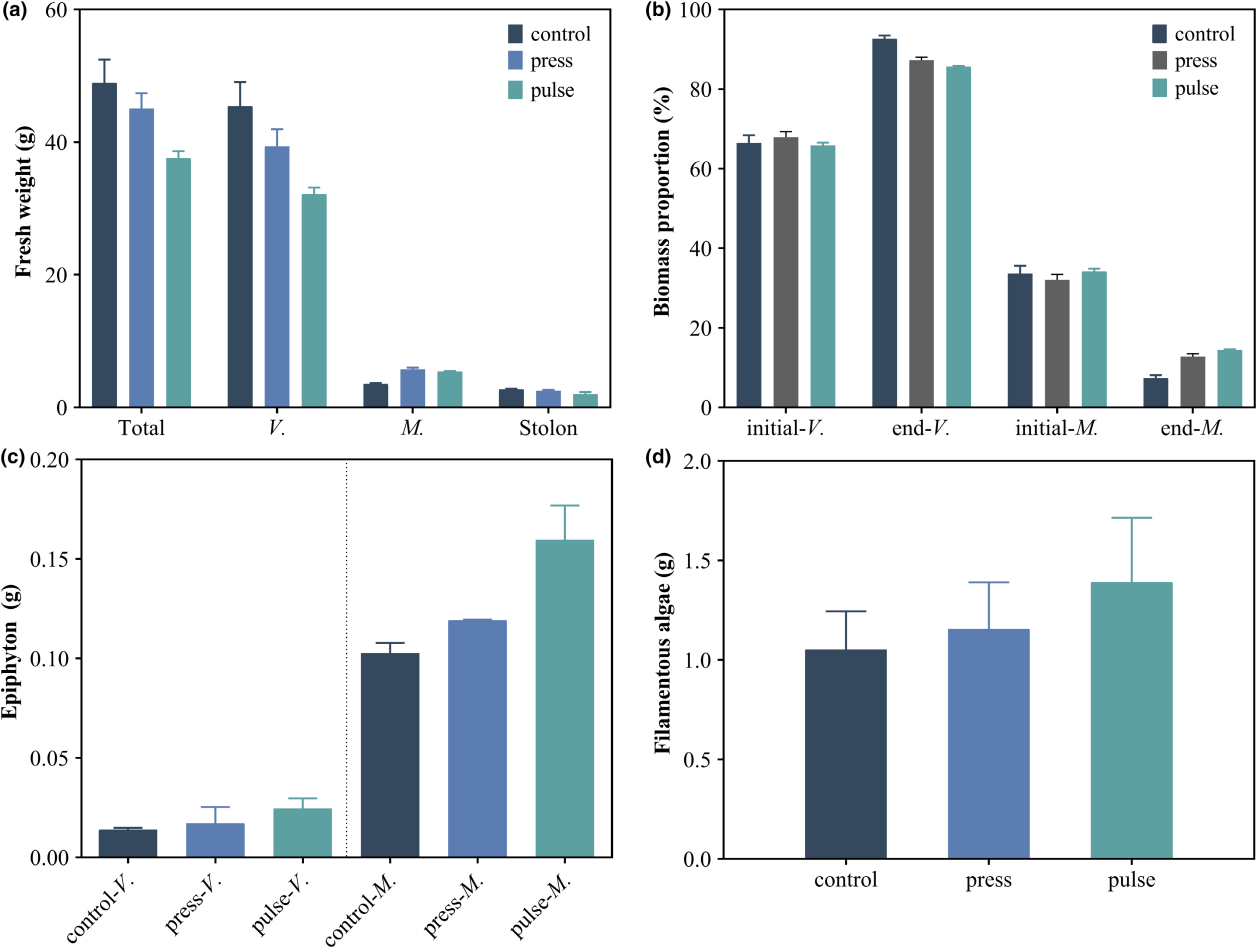
Changes in the fresh weight, biomass proportion, epiphyton, and filamentous algae. (a) Total fresh weight of submerged macrophytes, fresh weight of *Vallisneria natans* (*V*.), fresh weight of *Myriophyllum spicatum* (*M*.), and fresh weight of stolon for different treatments. (b) Biomass proportion of *V. natans* (*V*.) and *M. spicatum* (*M*.) in different treatments at the beginning and end of the experiment. (c) Epiphyton biomass attached to submerged macrophytes. (d) Filamentous algae biomass attached to the containers. The error bars represent standard error.

The total fresh weight of submerged macrophytes was the greatest in the control treatment, with a mean value of 48.93 ± 6.11 g, followed by the press treatment, with a mean value of 45.06 ± 3.28 g, and the lowest in the pulse treatment, with a mean value of 37.55 ± 1.87 g. The fresh weight of *V. natans* was the greatest in the control treatment, while it was the lowest in the pulse treatment. The fresh weight of *M. spicatum* was the lowest in the control treatment. The stolon fresh weight of *V. natans* was the greatest in the control treatment, with a mean value of 2.74 ± 0.13 g, while it was the lowest in the pulse treatment, with a mean value of 1.99 ± 0.55 g. The fresh weight of *V. natans* and stolon showed the same trend in the different treatments. The more stolon there was, the higher the biomass of *V. natans* (Figure 3a). The pulse treatment, characterized by a high nutrient concentration and algal density, had a low total fresh weight and *V. natans* fresh weight.

At the end of the experiment, the biomass proportion of *V. natans* increased in all treatments, while the proportion of *M. spicatum* decreased in all treatments (Figure 3b). The biomass proportion of *V. natans* was the greatest in the control treatment, with a mean value of 92.67 ± 1.34%, followed by the press treatment, with a mean value of 87.25 ± 1.29%, and the lowest in the pulse treatment, with a mean value of 85.60 ± 0.35%. The biomass proportions of *V. natans* and *M. spicatum* were significantly negatively correlated (*p* < .05). The treatment with a high proportion of *M. spicatum* had a low proportion of *V. natans*, implying that *M. spicatum* may have a competitive advantage over *V. natans*.

Due to the leaf morphology, the number of epiphytes attached to *M. spicatum* was higher than the number attached to *V. natans* in both treatments. Regardless of the type of submerged macrophyte, epiphyton biomass was higher in the pulse treatment than in the press treatment (Figure 3c). Filamentous algae biomass was the greatest in the pulse treatment, intermediate in the press treatment, and the lowest in the control treatment. The mean filamentous algae biomass was 1.05 ± 0.33 g in the control treatment, 1.16 ± 0.33 g in the press treatment, and 1.39 ± 0.56 g in the pulse treatment (Figure 3d). The biomasses of both the epiphyton and filamentous algae in the pulse treatment were higher than those in the press treatment. This showed the same trend as that for the TP concentration in the water column, which indicated that an increase in the TP concentration could promote the growth of epiphyton and filamentous algae.

**FIGURE 4:**
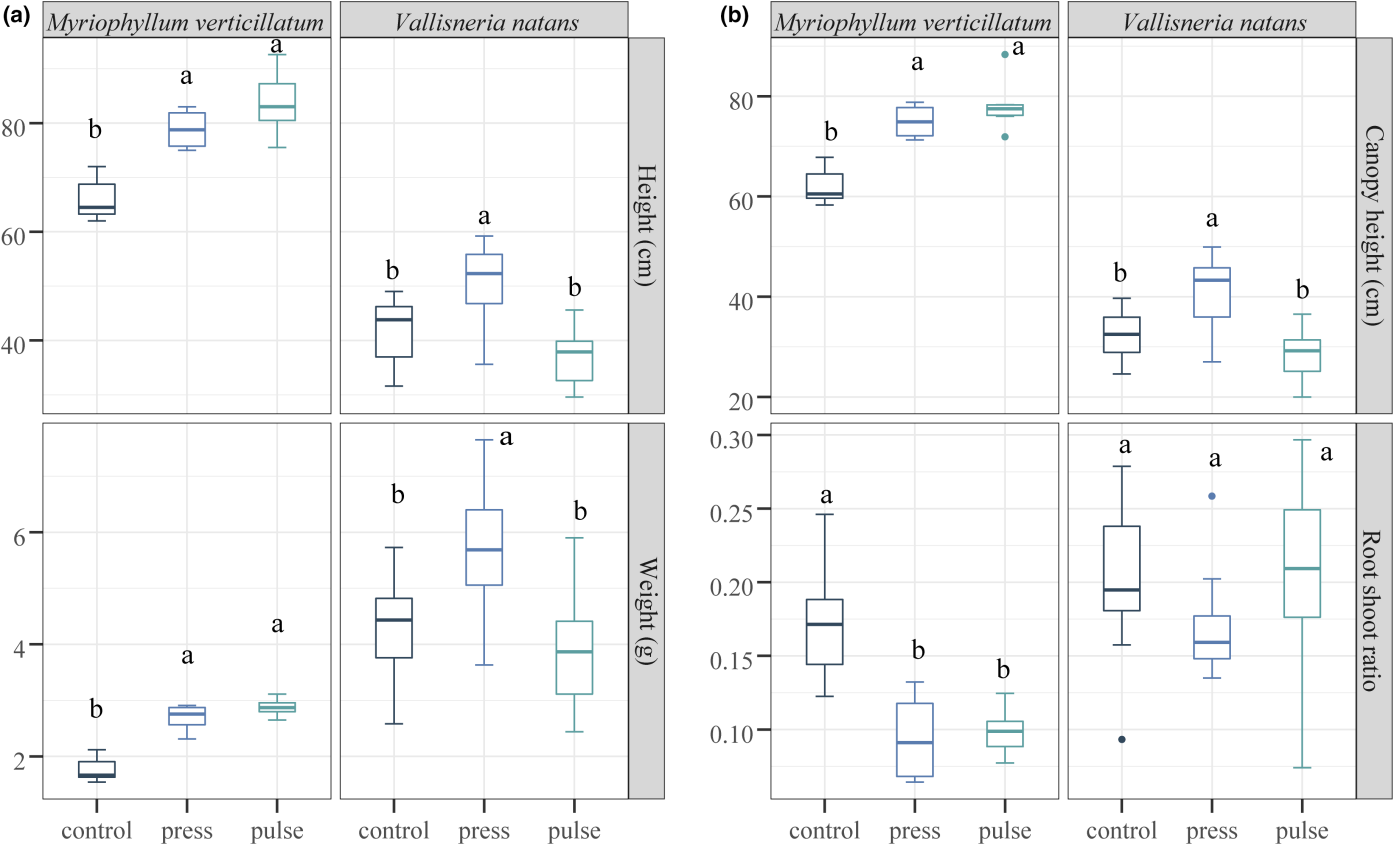
Morphological indicators of submerged macrophytes. (a) Plant height and plant weight of *Vallisneria natans* and *Myriophyllum spicatum* in different treatments. (b) Canopy height and root/shoot ratio of *V. natans* and *M. spicatum* in different treatments. The lowercase letters at the top of the boxplots indicate significant differences obtained by Tukey's least significant difference test at *p* < .05.

The plant height, plant weight, and canopy height of *V. natans* were significantly higher in the press treatment than in the remaining treatments (*p* < .05; Figure 4; Table S2). The plant height, weight, and canopy height of *M. spicatum* were significantly higher in the press and pulse treatments than in the control treatment (*p* < .05). The root/shoot ratio of *M. spicatum* was significantly higher in the control treatment than in the remaining treatments (*p* < .05) (Figure 4).

**FIGURE 5:**
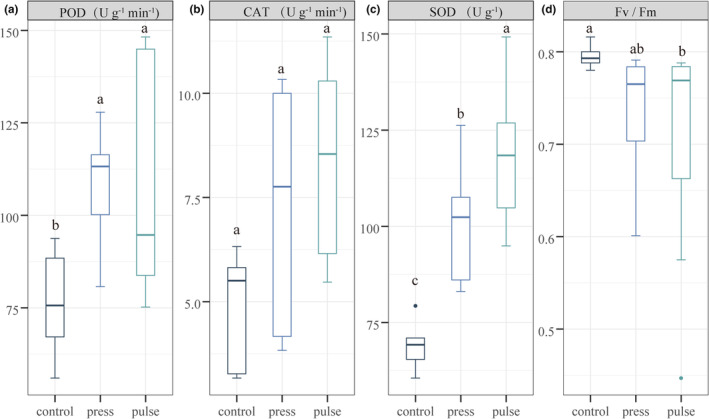
(a) Peroxidase (POD), (b) catalase (CAT), and (c) superoxide dismutase (SOD) of *Vallisneria natans* in different treatments. (d) The light energy conversion efficiency (*F*_v_/*F*_m_) of *V. natans* in different treatments. The lowercase letters at the top of the boxplots indicate significant differences obtained by Tukey's least significant difference test at *p* < .05.

When exposed to environmental stress, plants adapt to the pressure by enhancing their enzymatic activity. The POD and SOD enzyme contents of *V. natans* in the press and pulse treatments were significantly higher than those in the control treatment (*p* < .05; Figure 5a–c; Table S3). The mean CAT enzyme content of the plants was the highest in the pulse treatment, but there was no significant difference between treatments (*p* > .05). The SOD enzyme contents of the plants in the pulse treatment were significantly higher than those in the press treatment (*p* < .05).

*F*_v_/*F*_m_ reflects the maximum photosynthetic capacity of a plant, and its value decreases when plants are light‐limited. The *F*_v_/*F*_m_ of *V. natans* was 0.79 ± 0.01 in the control treatment, 0.73 ± 0.07 in the press treatment, and 0.69 ± 0.12 in the pulse treatment. The *F*_v_/*F*_m_ was significantly higher in the control treatment than in the pulse treatment (*p* < .05; Figure 5d).

### 3.3 Stability of the experimental aquatic ecosystem

**FIGURE 6:**
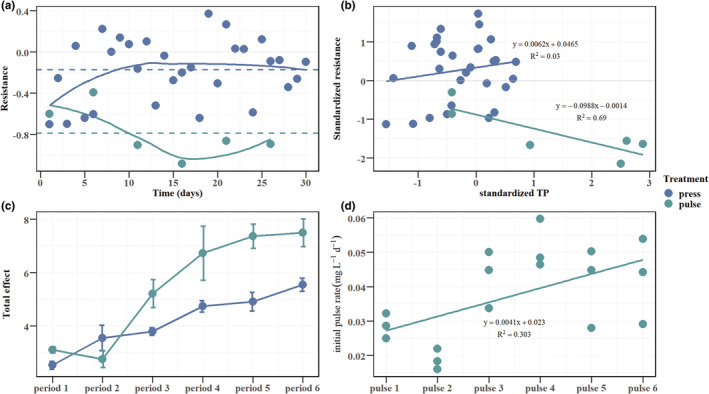
(a) Variation in resistance with time for different treatments. The curves were drawn by loess regression (locally weighted regression). The dotted lines represent the mean values. (b) Variation in resistance with TP for different treatments. (c) The total press effect and the total pulse effect in different periods. (d) The initial pulse rate of the pulse treatment for the six pulse additions.

Resistance was significantly higher in the press treatment than in the pulse treatment (*p* < .05). The resistance of the system to pulse disturbance decreased with time, whereas the resistance of the system to press disturbance was relatively stable (Figure 6a). As TP rose, the resistance of the system to the pulse disturbance decreased significantly (Figure 6b). Except for in period 2, the total pulse effect was greater than the total press effect, and the initial pulse rate showed an upwards tendency (Figure 6c,d).

## 4 DISCUSSION

Several studies have shown that aquatic systems respond differently to press and pulse disturbances (Parkyn & Collier, 2004; Urrutia‐Cordero et al., 2021). Systems receiving nutrient pulses tend to show a domed‐curve dynamic due to the rapid response of the primary producers in those ecosystems (Holt, 2008). The concentration of nutrients tends to drop to pre‐pulse levels after pulse disturbances (Yang et al., 2010). In our experiment, the TP concentration rose and then fell during every period in the pulse treatment. However, the TP concentration of the pulse treatment did not decrease to the pre‐pulse levels (Figure 2a), especially at a high TP concentration level, which may be due to a decrease in the system stability as the nutrient concentration and algal density increased (Figure 6). This suggests that pulse disturbances may pose a greater threat to lake ecosystems, especially in the context of eutrophication. Systems receiving continuous nutrient inputs tend to show smooth increases in nutrient concentrations. Our experiments showed an overall upwards trend in TP concentration and algal density in the press treatment (Figure 2a,c).

Differences in total phosphorus concentrations and algal density were observed between treatments in our study. The TP concentration and algal density of the water column were significantly higher in the pulse treatment than in the press treatment (*p* < .05), although both treatments received the same nutrient loads (Figure 2b,d). These differences in response indicated that the resistance varied between treatments. The analysis showed that the resistance was significantly higher in the press treatment than in the pulse treatment (*p* < .05; Figure 6a). The pulse treatment showed lower resistance to disturbances, especially at a higher nutrient concentration, indicating that lake ecosystems may show lower resistance to pulse disturbances as those ecosystems deteriorate (Dai et al., 2013). Both the total pulse effect and the initial pulse rate showed an upwards trend with time, which could be explained by the reduced resistance of the ecosystem as nutrients increased (Figure 6).

The different life strategies and abilities of algae and submerged macrophytes to exploit nutrients could explain these differences (Kautsky, 1988; Reynolds, 1987). The algae grow fast and have a high metabolism rate and nutrient requirements, but their biomass is often nutrient‐limited (Sand‐Jensen & Borum, 1991). Hence, this may have resulted in the higher algal density in the treatment with pulse nutrient inputs, although the press treatment received equal loads during the experiment. Submerged macrophytes grow more slowly compared to algae. The nutrient requirements of submerged macrophytes are lower than algae because of their low metabolism (Sand‐Jensen & Borum, 1991). In summary, the different nutrient uptake strategies of submerged macrophytes and algae resulted in higher TP concentrations and algal densities in the pulse treatment.

In our study, pulse treatment with higher TP concentrations and algal densities had lower total biomass of submerged macrophytes than that in the press treatment (Figures 2c and 3a). Submerged macrophytes are important for the resistance of aquatic ecosystems when facing disturbances (Moss, 1990). Studies have shown that shallow freshwater lakes with higher submerged plant biomass could facilitate a clear‐water state through nutrient uptake, allelopathic interactions, providing refuge and shading (Burks et al., 2002; Gross, 2003; Hector et al., 2001; Vandonk et al., 1993). There was positive feedback between the biomass of submerged macrophytes and water quality, contributing to the high resistance of the press disturbance‐receiving systems. Differences in community biomass are caused by changes in the biomass of individual species, which could show phenotypic adaptations to disturbances. In response to the poor light conditions in the press treatment, *V. natans* can physiologically adapt to meet its light requirements by elevating its canopy (Chou et al., 2022; Choudhury et al., 2019; Jiang et al., 2018; Li et al., 2020; Yang et al., 2022). However, the *V. natans* in the pulse treatment did not show a higher canopy height or plant height than the *V. natans* in the press treatment (Figure 4). This may be because *V. natans* was subjected to more severe stress and lost its ability to make morphological adaptations during the pulse disturbance (Qi et al., 2018).

In addition to morphological indicators, physiological indicators can be used to assess the resistance of submerged macrophytes to disturbances. The inhibition of *V. natans* in the pulse treatment was also evidenced by its lower light conversion efficiency (*F*_v_/*F*_m_) and higher enzyme content (Figure 5; Cao et al., 2004; Marwood et al., 2001; Wang et al., 2016). In contrast, *M. spicatum*, a eutrophic species, is more resistant to nutrient disturbances. The plant height, canopy height, and plant weight of *M. spicatum* were significantly higher in the press and pulse treatments than in the control treatment (Figure 4). Furthermore, plants allocate more resources to shoot development than to root development under higher nutrient and light‐limited condition. And they will show a lower root/shoot ratio (Weiner, 2004). The lower root/shoot ratio of *M. spicatum* in the press and pulse treatments indicated a poor light environment (Figure 4). As nutrient increases, the number of epiphyton present on *M. spicatum* may increase, which could affect the plant's photosynthesis and cause mortality (Accoroni et al., 2017; Wijewardene et al., 2022).

There are increasing concerns about disturbances to aquatic ecosystems, which could change the structure and function of ecosystems (Moller et al., 2015; Rabalais et al., 2009; Verhoeven et al., 2006). Studies have revealed that the input of nutrients leads to an increase in primary productivity (Gende et al., 2002; Naiman et al., 2002; Nowlin et al., 2008). We found that both press and pulse nutrient inputs increased algal density. Weber and Brown (2013) found that biotic and abiotic indicators tended to be less stable in systems experiencing pulse inputs of nutrients but more stable in systems experiencing press inputs of nutrients. Our findings are consistent with that study. The system that received pulse disturbances exhibited instability, reflected in its lower resistance (Figure 6). Natural conditions are often more complex than experimental conditions, with disturbances varying in intensity and frequency. Our experiments, however, were conducted in a microcosmic aquatic system and involved only two forms of disturbances. Larger‐scale experiments with multiple proxy indicators deserve to be carried out further. In addition, as climate change and incidents of extreme events increase, press and pulse disturbances will co‐occur. Whether the interaction between press disturbances and pulse disturbances will cause more serious ecological consequences should be considered in the future.

## 5 CONCLUSION

In this study, we demonstrated the differences in total phosphorus, algae density, and physiological indicators of macrophytes in the experimental aquatic ecosystems under press and pulse nutrient disturbances. The results showed that pulse disturbance‐receiving systems had a higher TP concentration, algae density, and lower resistance than press disturbance‐receiving systems. The submerged macrophyte communities were suppressed in the pulse treatment, as evidenced by the lowest biomass. It is also important to note that various kinds of disturbances may become more frequent due to climate change and the effects of disturbance on aquatic ecosystems deserve to be further explored.

## AUTHOR CONTRIBUTIONS

**Yu Zhao:** Conceptualization (equal); investigation (equal); writing – original draft (equal). **Rong Wang:** Supervision (equal); writing – review and editing (equal). **Enlou Zhang:** Writing – review and editing (equal). **Baohua Guan:** Methodology (equal); visualization (equal). **Min Xu:** Methodology (equal); visualization (equal).

## CONFLICT OF INTEREST

The authors declare no competing interests.

## Supporting information


Figure S1

Tables S1–S3

## Data Availability

Data used in this study are available at the Dryad Digital Repository (https://doi.org/10.5061/dryad.pzgmsbcqn).
