# Supplementary figures and images for: Aquatic ecosystem responds differently to press and pulse nutrient disturbances as revealed by a microcosm experiment
Source: Ecol Evol. 2022 Oct 22;12(10):e9438. doi: 10.1002/ece3.9438 (PMC9587460; doi:10.1002/ece3.9438)

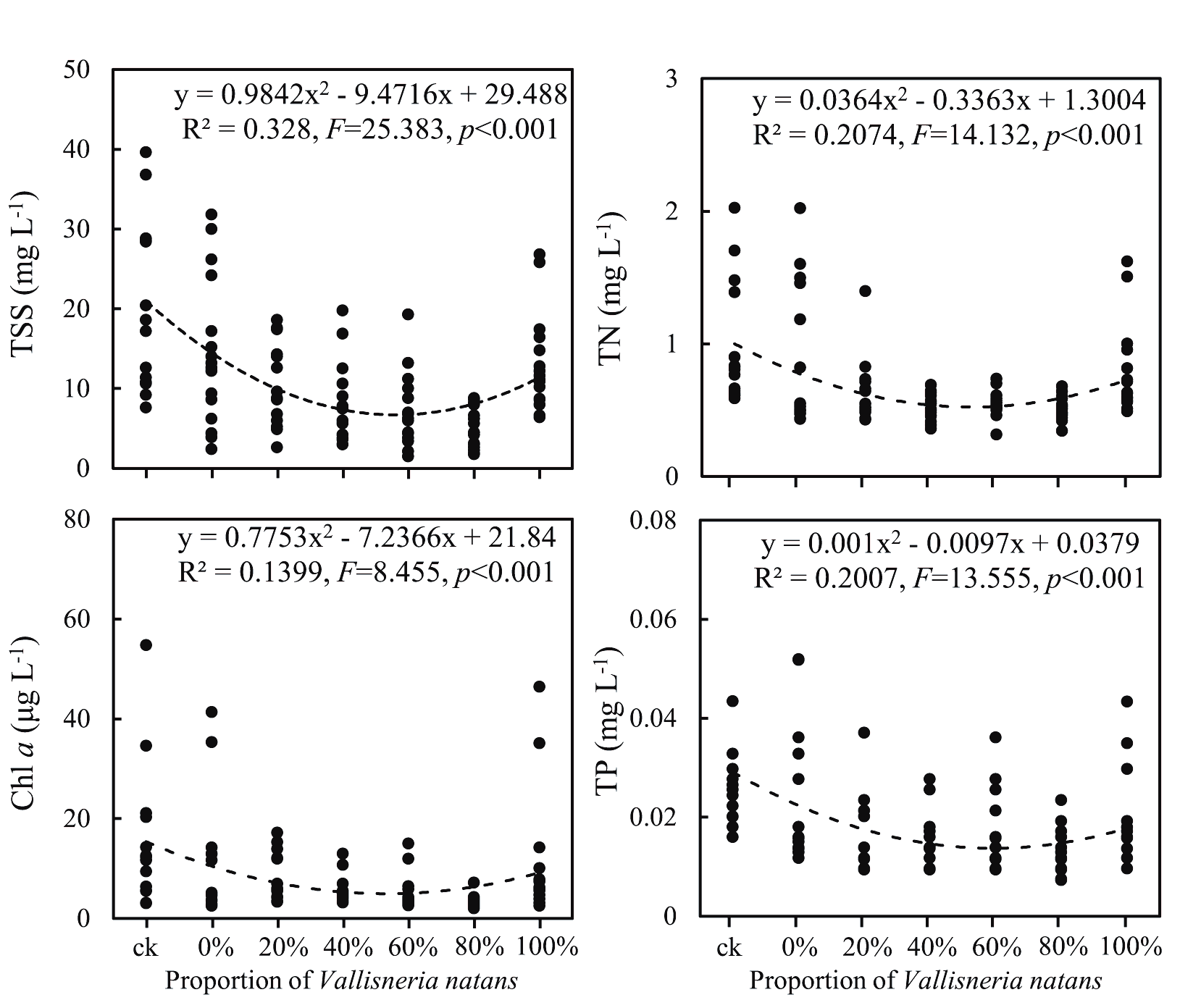

Supplement: Supplementary file 1 — Figure S1 [file ECE3-12-e9438-s001.tif]
