# Supplementary material for: Aquatic ecosystem responds differently to press and pulse nutrient disturbances as revealed by a microcosm experiment
Source: Ecol Evol. 2022 Oct 22;12(10):e9438. doi: 10.1002/ece3.9438 (PMC9587460; doi:10.1002/ece3.9438)
Supplement: Supplementary file 2 — Tables S1–S3 [file ECE3-12-e9438-s002.docx]

# Appendix

**Table S1**. Summary of one-way ANOVA results for the concentrations of TP and OD680 of different treatments.

|  | | df | F | *P* |  |
| --- | --- | --- | --- | --- | --- |
| TP | | 2 | 115.938 | <0.001 |  |
| OD680 | | 2 | 78.693 | <0.001 |  |
| Post hoc multiple comparisons | | | | | |
| LSD | （I）treatment | （J）treatment | mean difference  （I-J） | *P* |  |
| TP | control | press | -.012 | <0.001 |  |
|  | control | pulse | -.030 | <0.001 |  |
|  | press | pulse | -.018 | <0.001 |  |
| OD680 | control | press | -.044 | <0.001 |  |
|  | control | pulse | -.088 | <0.001 |  |
|  | press | pulse | -.044 | <0.001 |  |

**Table S2**. Summary of one-way ANOVA results for the height, canopy height, weight and root shoot ratio of *Vallisneria natans* and *Myriophyllum spicatum* in different treatments*.*

| Factor | | Species | df | F | *P* |
| --- | --- | --- | --- | --- | --- |
| Height | | *Vallisneria natans* | 2 | 10.805 | <0.001 |
|  |  | *Myriophyllum spicatum* | 2 | 13.339 | .001 |
| Canopy height | | *Vallisneria natans* | 2 | 10.287 | <0.001 |
|  |  | *Myriophyllum spicatum* | 2 | 15.165 | <0.001 |
| Weight | | *Vallisneria natans* | 2 | 4.848 | .015 |
|  |  | *Myriophyllum spicatum* | 2 | 38.631 | <0.001 |
| Root shoot ratio | | *Vallisneria natans* | 2 | 1.389 | .263 |
|  |  | *Myriophyllum spicatum* | 2 | 9.116 | .003 |
| Post hoc multiple comparisons | | | | | |
| LSD | | （I）treatment | （J）treatment | mean difference  （I-J） | *P* |
| *Vallisneria natans* | Height | control | press | -8.050 | .023 |
|  |  | control | pulse | 5.258 | .118 |
|  |  | press | pulse | 13.308 | <0.001 |
|  | Canopy height | control | press | -8.046 | .017 |
|  |  | control | pulse | 4.300 | .200 |
|  |  | press | pulse | 12.346 | <0.001 |
|  | Weight | control | press | -1.282 | .022 |
|  |  | control | pulse | .321 | .504 |
|  |  | press | pulse | 1.603 | .005 |
| *Myriophyllum spicatum* | Height | control | press | -16.600 | .001 |
|  |  | control | pulse | -12.000 | .005 |
|  |  | press | pulse | .156 | .537 |
|  | Canopy height | control | press | -16.575 | .001 |
|  |  | control | pulse | -12.067 | .003 |
|  |  | press | pulse | 4.508 | .376 |
|  | Weight | control | press | -1.092 | <0.001 |
|  |  | control | pulse | -.937 | <0.001 |
|  |  | press | pulse | .156 | .537 |
|  | Root shoot ratio | control | press | .0748 | .011 |
|  |  | control | pulse | .0739 | .006 |
|  |  | press | pulse | <-.001 | .999 |

**Table S3**. Summary of one-way ANOVA results for the concentrations of POD, CAT, SOD, and *Fv*/*Fm* of different treatments.

|  | | df | F | *P* |
| --- | --- | --- | --- | --- |
| POD | | 2 | 4.192 | .042 |
| CAT | | 2 | 2.526 | .121 |
| SOD | | 2 | 15.096 | <0.001 |
| Fv/Fm | | 2 | 5.105 | .012 |
| Resistance | | 1 | 3.978 | .003 |
| Post hoc multiple comparisons | | | | |
| LSD | （I）treatment | （J）treatment | mean difference  （I-J） | *P* |
| POD | control | press | -31.477 | .031 |
|  | control | pulse | -33.161 | .025 |
|  | press | pulse | -1.684 | .898 |
| SOD | control | press | -30.740 | .003 |
|  | control | pulse | -48.879 | <0.001 |
|  | press | pulse | -18.140 | .040 |
| *Fv*/*Fm* | control | press | .057 | .064 |
|  | control | pulse | .088 | .004 |
|  | press | pulse | .031 | .312 |

**
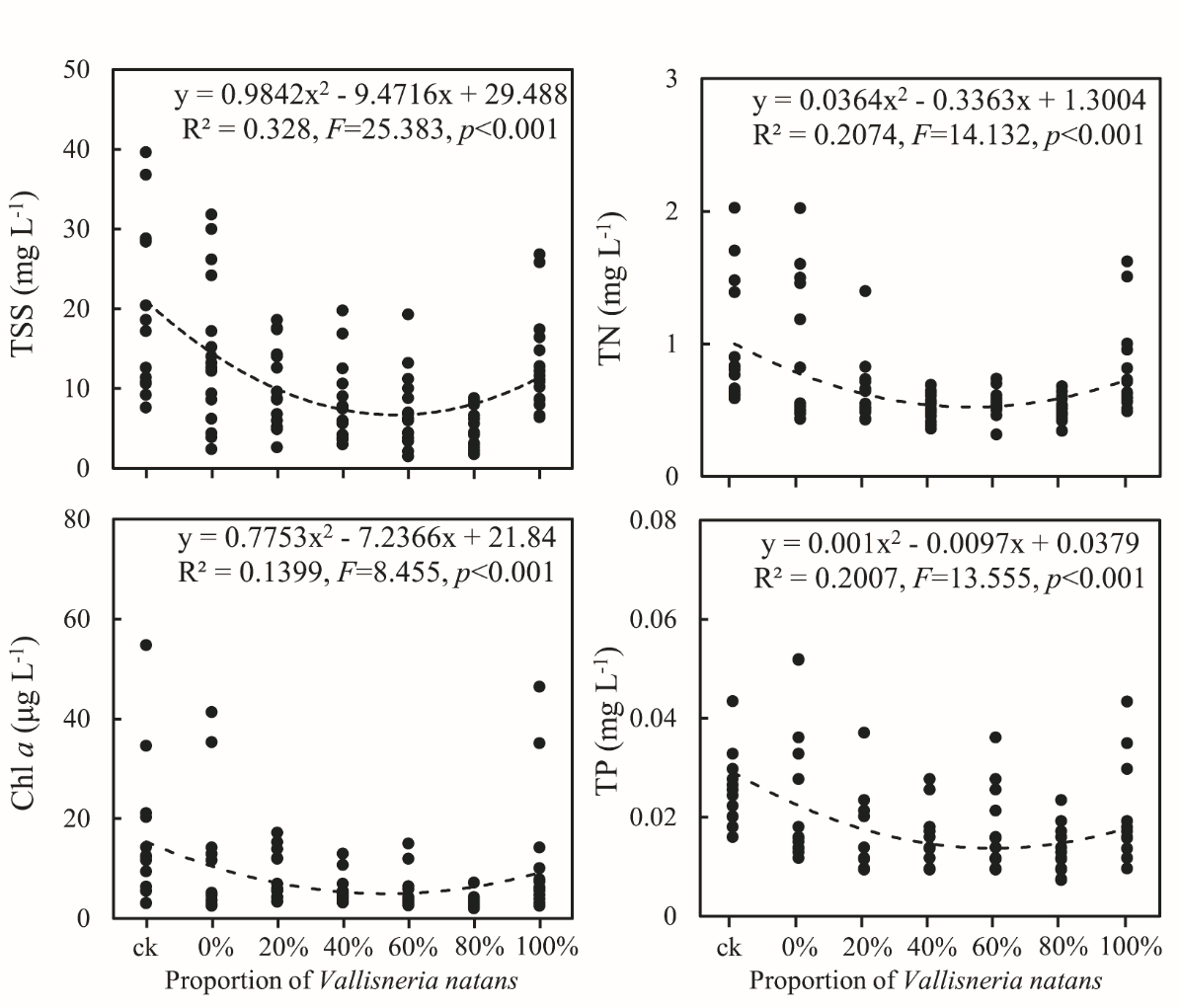
**

**Fig. S1.** Changes of water quality with the increase of the proportion of *Vallisneria natans.* A total of two submerged macrophytes, *V. natans* and *Myriophyllum spicatum*, were planted. Ck represents the treatment with no plants. The concentrations of the total suspended solids (TSS), total nitrogen (TN), chlorophyll *a* (Chl *a*), and total phosphorus (TP) in the water column were measured. The water quality was maintained in optimal clear states when the biomass proportions of *V. natans* were between 60 % and 80 % in the communities.
